# Um Achado Raro: Caso de Angioma Cardíaco em Paciente Adulto

**DOI:** 10.36660/abc.20240150

**Published:** 2024-12-11

**Authors:** Inês Conde, Rodrigo Silva, Nuno Antunes, Vitor Hugo Pereira, Catarina Quina-Rodrigues

**Affiliations:** 1 Hospital de Braga Braga Portugal Hospital de Braga, Braga – Portugal

**Keywords:** Hemangioma, Ecocardiografia, Técnicas de Imagem Cardíaca

## Introdução

As massas cardíacas abrangem um amplo espectro de etiologias, incluindo tumores primários, tanto benignos quanto malignos, tumores metastáticos e lesões não neoplásicas.^1,2^ O diagnóstico diferencial das massas cardíacas é desafiador devido à sobreposição de apresentações clínicas, características de imagem diversificada, heterogeneidade entre diferentes tipos de massa, experiência clínica limitada e raridade de algumas dessas condições.^1,2^ O presente caso clínico apresenta um cenário intrigante envolvendo uma massa cardíaca, que destaca as complexidades no seu diagnóstico e manejo.

## Apresentação do caso

**Figura 1:**
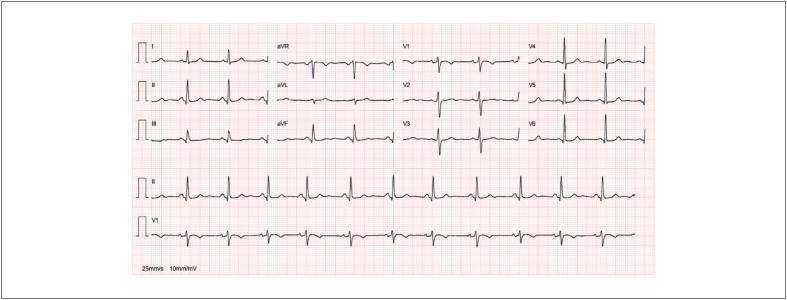
Eletrocardiograma mostrando ritmo sinusal sem alterações significativas.

**Figura 2:**
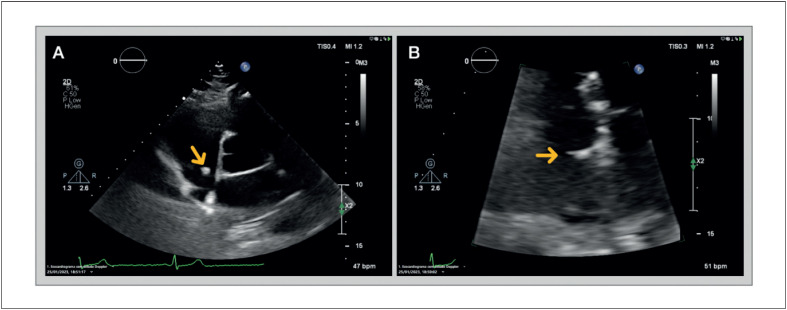
Ecocardiograma transtorácico PSAX ao nível da valva aórtica (A) e apical 4 câmaras (B; ampliado), evidenciando massa arredondada na aurícula direita, ecogênica, móvel, de etiologia duvidosa, com cerca de 6 mm de diâmetro, em possível relação com a válvula de Eustáquio.

**Figura 3:**
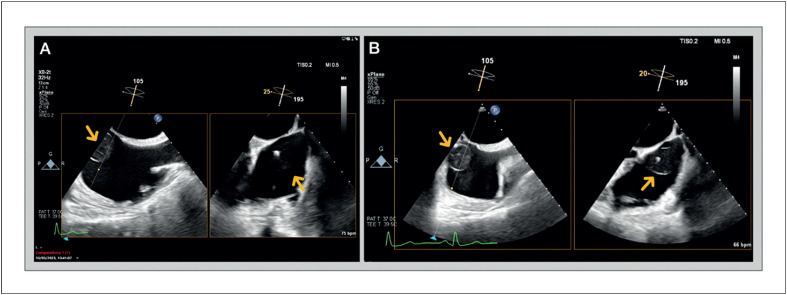
Ecocardiograma transesofágico, corte médio esofágico bicaval e plano X (A) e (B), evidenciando, ao nível da saída da veia cava inferior, volumosa massa arredondada (25×32 mm), pediculada, com limites bem definidos e aspecto capsulado (bordas ecogênicas com interior sólido aparentemente hipoecogênico em relação às paredes cardíacas).

**Figura 4:**
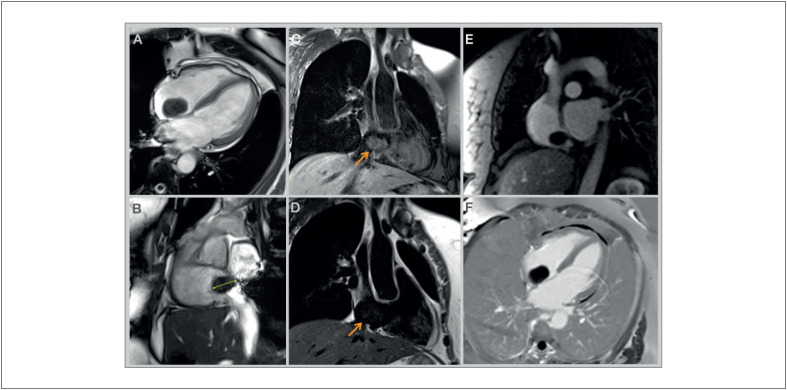
Ressonância magnética cardíaca revelando massa móvel na aurícula direita, bem delimitada (A), com 28 × 15 × 25 mm (B) de tamanho, aparentemente oriunda da porção inferior do septo interatrial. A referida massa era isointensa em T1 (C), isointensa em T2 (D), sem perfusão nas imagens de primeira passagem (E) e sem realce precoce ou tardio (F).

**Figura 5:**
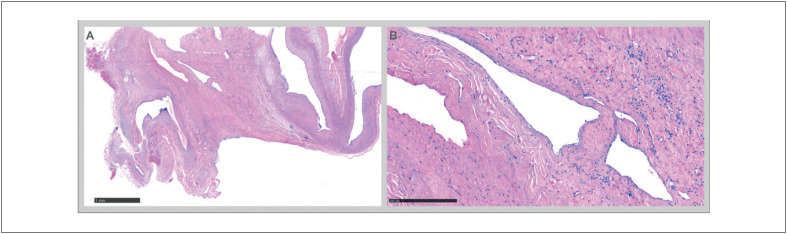
Análise histopatológica do tecido removido, estabelecendo o diagnóstico de angioma cardíaco: (A) Lesão angiomatosa de vasos ectásicos; o miocárdio pode ser visto na periferia. (B) Vasos revestidos por células endoteliais sem atipias.

Paciente do sexo feminino, 60 anos de idade, com histórico de diabetes tipo 2 e dislipidemia, foi encaminhada ao Ambulatório de Cardiologia devido a quadro de dor torácica opressiva e não relacionada a esforços nos últimos meses. Ela não apresentava outras queixas, como dispneia, edema periférico, síncope, palpitações, febre, perda de peso ou fadiga. Seu exame físico era normal. O eletrocardiograma (Figura 1) apresentava ritmo sinusal, sem alterações significativas. O exame de sangue mostrou hemoglobina, função renal e tireoidiana normais. O ecocardiograma transtorácico (Figura 2) revelou câmaras cardíacas de tamanho normal, espessura da parede ventricular normal e função sistólica biventricular preservada. No átrio direito, foi identificada uma massa arredondada, ecogênica e móvel, de etiologia incerta, aparentemente oriunda da válvula de Eustáquio, medindo aproximadamente 6 mm de diâmetro. Diante dessa constatação, novos testes foram solicitados. O ecocardiograma transesofágico (Figura 3) mostrou, ao nível da veia cava inferior, grande massa arredondada (25×32 mm), pediculada, com bordas bem definidas e aspecto capsulado (bordas ecogênicas com interior sólido aparentemente hipoecogênico, comparado às paredes cardíacas). A ressonância magnética cardíaca (RMC; Figura 4) confirmou a presença de massa móvel, bem delimitada, com 28×15×25 mm de tamanho, aparentemente oriunda da porção inferior da face direita do septo interatrial. A referida massa era isointensa em T1, isointensa em T2, sem perfusão nas imagens de primeira passagem e sem realce precoce ou tardio. Apesar da ausência de hipersinal nas imagens ponderadas em T2, esses achados poderiam ser compatíveis com o diagnóstico de mixoma, mas não foi possível estabelecer um diagnóstico definitivo. Após discussão multidisciplinar, o paciente foi encaminhado para excisão cirúrgica da massa. A ressecção cirúrgica foi bem-sucedida e o pós-operatório transcorreu sem complicações. A análise histopatológica do tecido removido permitiu o diagnóstico definitivo de angioma cardíaco (Figura 5).

## Discussão

O diagnóstico diferencial das massas cardíacas apresenta desafios significativos devido à diversidade de patologias que podem acometer o coração.^1,2^ Sintomas comuns incluem dor torácica, palpitações, dispneia e sinais de insuficiência cardíaca. No entanto, alguns pacientes podem permanecer assintomáticos e a massa pode ser descoberta incidentalmente durante exames de imagem cardíacos solicitados por outros motivos.^1^

A diferenciação entre massas benignas e malignas, bem como a identificação de tipos específicos de tumores, é fundamental para o manejo adequado e obtenção de resultados satisfatórios para o paciente.^2,3^ Embora as técnicas de imagem, como ecocardiografia, RMC e tomografia computadorizada, desempenhem um papel crucial na avaliação inicial das massas cardíacas, elas podem nem sempre garantir uma diferenciação definitiva entre lesões benignas e malignas, ou tipos específicos de tumores.^4,5^ A análise histopatológica de uma amostra de tecido, obtida por meio de ressecção cirúrgica ou biópsia, desempenha um papel central na obtenção de um diagnóstico preciso, orientando as decisões de tratamento e otimizando o atendimento ao paciente.^6,7^

O angioma cardíaco, também conhecido como hemangioma cardíaco, é um tumor benigno raro que se origina nos vasos sanguíneos do coração.^8^ Este tumor cresce lentamente e frequentemente regride espontaneamente durante a infância.^8^ A prevalência exata e a distribuição etária dos angiomas cardíacos não estão bem estabelecidas devido à sua raridade e à limitação dos dados disponíveis. Contudo, diversos estudos e relatos de casos observaram maior incidência em crianças e lactentes. Devido à sua raridade, existem poucos estudos disponíveis com foco específico em casos na população adulta.^8-10^

Este tumor vascular raro pode ocorrer em qualquer camada do coração, incluindo endocárdio, miocárdio ou pericárdio. Embora se pensasse anteriormente que o átrio esquerdo era o local predominante para este tumor, evidências recentes indicam que não há predileção por câmara específica.^9^

Na RMC, os angiomas cardíacos geralmente aparecem como massas heterogêneas com intensidade de sinal moderada a alta nas imagens ponderadas em T1 e alto sinal difuso nas imagens ponderadas em T2. O agente de contraste geralmente é absorvido de forma desigual, com realce significativo durante a fase arterial, resultando em alta intensidade de sinal e realce prolongado.^11^ No caso apresentado aqui, os achados da RMC foram discordantes daqueles geralmente descritos nesse tipo de tumor. Considerando a idade avançada do nosso paciente, pode-se sugerir que esta massa seja um angioma antigo, que gradualmente perdeu vascularização, o que explicaria a ausência de hipersinal nas imagens ponderadas em T2 e a falta de perfusão nas imagens de primeira passagem. Além disso, a localização da massa favorecia mais a hipótese de mixoma em comparação com outros tumores, sendo nossa principal suspeita antes da excisão e dos resultados patológicos, mesmo sem hipersinal evidente nas imagens ponderadas em T2.

O manejo de pacientes requer uma abordagem multidisciplinar, frequentemente envolvendo cardiologistas, cirurgiões cardiotorácicos, radiologistas e patologistas. A experiência conjunta desses profissionais facilita decisões de tratamento bem fundamentadas, o planejamento da intervenção cirúrgica e garante cuidado integral ao paciente. A ressecção cirúrgica é a principal modalidade de tratamento para angiomas cardíacos. O objetivo da cirurgia é conseguir a excisão completa do tumor, preservando a função cardíaca.^10^ A técnica cirúrgica e a abordagem dependem da localização, tamanho e extensão do angioma no coração.^10^

Os angiomas cardíacos são tipicamente considerados tumores benignos, que geralmente apresentam prognóstico mais favorável em comparação com os tumores malignos.^8,9^ A extensão da ressecção cirúrgica desempenha um papel fundamental no prognóstico dos angiomas cardíacos.^8-10^ A remoção completa do tumor normalmente está associada a um melhor prognóstico, já que elimina o risco de recorrência e reduz o potencial de complicações.^9,10^ A ressecção incompleta ou tumor residual pode aumentar a probabilidade de recorrência ou exigir intervenções adicionais.

Após a remoção cirúrgica de angiomas cardíacos, é crucial realizar acompanhamento e vigilância regulares, incluindo avaliações periódicas por imagem para monitorar sinais de recorrência ou presença residual da doença, avaliar a função cardíaca e detectar possíveis complicações. A frequência e a duração do acompanhamento podem variar dependendo de características individuais do paciente e do tumor.^9^

É importante observar que, devido ao número limitado de casos e à raridade dos angiomas cardíacos, prever o prognóstico para um paciente individual pode ser desafiador. O prognóstico é influenciado por diversos fatores, como as características do tumor, o sucesso da remoção cirúrgica e a ausência de complicações. Assim, um monitoramento rigoroso, acompanhamento regular e cuidados multidisciplinares envolvendo cardiologistas, cirurgiões cardiotorácicos e patologistas são essenciais para garantir o manejo ideal e os resultados de longo prazo para pacientes com angiomas cardíacos.^7,9^

## Conclusão

Devido à sua raridade, as massas cardíacas frequentemente representam um desafio diagnóstico na prática clínica. Embora as técnicas de imagem sejam fundamentais para o diagnóstico de massas cardíacas, elas nem sempre proporcionam uma diferenciação definitiva entre lesões benignas e malignas ou tipos específicos de tumores. Muitas vezes, a única forma de estabelecer definitivamente um diagnóstico é por meio de análise histopatológica de uma amostra de tecido. Diagnóstico definitivo como implicação importante no manejo e prognóstico do paciente.
