# Supplementary material for: Um Achado Raro: Caso de Angioma Cardíaco em Paciente Adulto
Source: Arq Bras Cardiol. 2024 Dec 11;121(12):e20240150. [Article in Portuguese] doi: 10.36660/abc.20240150 (PMC11634299; doi:10.36660/abc.20240150)
Supplement: Translation

# Translation: A Rare Encounter: Case of Cardiac Angioma in an Adult Patient

**DOI:** 10.36660/abc.20240150i

**Authors:** Inês Conde, Rodrigo Silva, Nuno Antunes, Vitor Hugo Pereira, Catarina Quina-Rodrigues

**Affiliations:** 1 Braga Portugal Hospital de Braga, Braga – Portugal

**Keywords:** Hemangioma, Echocardiography, Cardiac Imaging Techniques

## Introduction

Cardiac masses encompass a wide spectrum of etiologies, including primary tumors, both benign and malignant, metastatic tumors, and non-neoplastic lesions.^1,2^ The differential diagnosis of cardiac masses is challenging due to overlapping clinical presentations, diverse imaging characteristics, heterogeneity among different mass types, limited clinical experience, and the rarity of some of these conditions.^1,2^ This clinical case presents an intriguing scenario involving a cardiac mass, highlighting the complexities in its diagnosis and management.

## Case presentation

A 60-year-old woman with a history of type 2 diabetes and dyslipidemia was referred to a Cardiology Outpatient Clinic due to oppressive chest pain unrelated to exertion over the previous months. She had no further complaints, namely dyspnea, peripheral edema, syncope, palpitations, fever, weight loss or fatigue. Her physical examination was unremarkable. Her electrocardiogram (Figure 1) showed sinus rhythm with no further changes. Blood work showed normal hemoglobin, renal and thyroid function. A transthoracic echocardiogram (Figure 2) revealed normally sized heart chambers, normal ventricular wall thickness and preserved biventricular systolic function. Within the right atrium, a rounded, echogenic, and mobile mass of uncertain etiology, apparently arising from the Eustachian valve, measuring approximately 6 mm in diameter, was identified. Given this finding, further testing was requested. A transesophageal echocardiogram (Figure 3) showed, at the level of the inferior vena cava, a large, rounded mass (25×32 mm), pediculated, with well-defined borders and capsulated aspect (echogenic borders with apparently hypoechogenic solid interior, compared to the cardiac walls). Cardiac magnetic resonance imaging (CMR; Figure 4) confirmed the presence of a well-delimited, mobile mass, 28×15×25 mm in size, with apparent origin in the lower portion of the right face of the interatrial septum. This mass was isointense on T1, isointense on T2, without perfusion on first-pass images and early or late enhancement. In spite of the absence of a hyper signal in T2-weighted images, these findings could be compatible with the diagnosis of myxoma, but a definitive diagnosis could not be established. After a multidisciplinary discussion, the patient was referred for surgical excision of the mass. Surgical resection was successful and the post-operative period was unremarkable. Histopathological analysis of the removed tissue allowed for the definitive diagnosis of a cardiac angioma (Figure 5).

**Figure 1:** Electrocardiogram showing sinus rhythm with no further changes.

**Figure 2:** Transthoracic echocardiogram PSAX at the level of the aortic valve (A) and apical 4 chambers (B; zoomed in), showing in the right auricle, in possible relation to the Eustachian valve, a rounded, echogenic, mobile mass of doubtful etiology, with about 6 mm in diameter.

**Figure 3:** Transesophageal echocardiogram mid-esophageal bicaval view and X-plane (A) and (B), showing, at the level of the outflow of the inferior vena cava, a voluminous rounded mass (25×32 mm), pediculated, with well-defined borders and capsulated aspect (echogenic borders with apparently hypoechogenic solid interior in relation to the cardiac walls).

**Figure 4:** Cardiac magnetic resonance showing, in the right auricle, a well delimited, mobile mass (A), 28 × 15 × 25 mm in size (B), with apparent origin in the lower portion of the interatrial septum. This mass was isointense on T1 (C), isointense on T2 (D), without perfusion on first-pass images (E) and without early or late enhancement (F).

**Figure 5:** Histopathological analysis of the removed tissue establishing the diagnosis of cardiac angioma: (A) Angiomatous lesion of ectatic vessels; myocardium can be seen on the periphery. (B) Vessels lined by endothelial cells without atypia.

## Discussion

The differential diagnosis of cardiac masses presents significant challenges due to the diverse range of pathologies that can affect the heart.^1,2^ Common symptoms include chest pain, palpitations, dyspnea, and signs of heart failure. However, some patients may remain asymptomatic, and the mass may be incidentally discovered during cardiac imaging for other reasons.^1^

Differentiating between benign and malignant masses, as well as identifying specific tumor types, is crucial for appropriate management and patient outcomes.^2,3^ While imaging techniques, such as echocardiography, CMR, and computerized tomography, play a crucial role in the initial evaluation of cardiac masses, they may not always provide definitive differentiation between benign and malignant lesions or specific tumor types.^4,5^ Histopathological analysis of a tissue sample obtained through surgical resection or biopsy plays a pivotal role in achieving an accurate diagnosis, guiding treatment decisions, and optimizing patient care.^6,7^

Cardiac angioma, also known as cardiac hemangioma, is a rare benign tumor that originates from blood vessels in the heart.^8^ This tumor grows slowly and frequently regresses spontaneously during childhood.^8^ The exact prevalence and age distribution of cardiac angiomas are not well established due to their rarity and the limited data available. However, several studies and case reports have reported a higher incidence in children and infants. Due to its rarity, there is limited literature available specifically focusing on adult cases.^8-10^

This rare vascular tumor can occur in any layer of the heart, including the endocardium, myocardium, or pericardium. While the left atrium was previously thought to be the predominant site for this tumor, recent evidence indicates that there is no specific chamber predilection.^9^

On CMR, cardiac angiomas typically appear as heterogeneous masses with moderate to high signal intensity on T1-weighted images and a diffuse high signal on T2-weighted images. The contrast agent is usually absorbed unevenly, with significant enhancement during the arterial phase, resulting in high signal intensity and prolonged enhancement.^11^ In our case, CMR findings were discordant with those usually described in this type of tumor. Given our patient's advanced age, it can be suggested that this mass was an ancient angioma that became progressively less vascularized, thus justifying the absence of hyper signal in T2-weighted images and lack of perfusion on first pass images. In addition, the location of the mass was more suggestive of a myxoma rather than another tumor, and this was our first hypothesis before the excision and pathological findings, even in the absence of hyper signal in T2-weighted images.

Patient management requires a multidisciplinary team approach, typically involving collaboration among Cardiologists, Cardiothoracic Surgeons, Radiologists, and Pathologists. Their combined expertise helps in making informed treatment decisions, planning the surgical approach, and ensuring comprehensive patient care. Surgical resection is the primary treatment modality for cardiac angiomas. The goal of surgery is to achieve complete excision of the tumor while preserving cardiac function.^10^ The surgical technique and approach depend on the location, size, and extent of the angioma within the heart.^10^

Cardiac angiomas are typically considered benign tumors, which generally have a more favorable prognosis compared to malignant tumors.^8,9^ The extent of surgical resection plays a crucial role in the prognosis of cardiac angiomas.^8-10^ Complete removal of the tumor is typically associated with a better prognosis, as it eliminates the risk of recurrence and reduces the potential for complications.^9,10^ Incomplete resection or residual tumor may increase the likelihood of recurrence or require additional interventions.

Regular follow-up and surveillance are important after surgical resection of cardiac angiomas. These include periodic imaging evaluations to monitor for signs of recurrence or residual disease, assess cardiac function, and detect potential complications. The frequency and duration of follow-up may vary depending on individual patient factors and the characteristics of the tumor.^9^

It is important to note that due to the limited number of cases and the rarity of cardiac angiomas, predicting the prognosis for an individual patient can be challenging. Various factors, including tumor characteristics, the success of surgical resection, and the absence of complications influence the prognosis. Therefore, close monitoring, regular follow-up, and multidisciplinary care involving cardiologists, cardiothoracic surgeons, and pathologists are crucial in ensuring optimal management and long-term outcomes for patients with cardiac angiomas.^7,9^

## Conclusion

Due to their rarity, cardiac masses often present a diagnostic challenge in clinical practice. Although imaging techniques are essential in the diagnosis of cardiac masses, they may not always provide definitive differentiation between benign and malignant lesions or specific tumor types. Often, the only way to definitively establish a diagnosis is through histopathological analysis of a tissue sample. Definitive diagnosis has important implications for the patient's management and prognosis.
